# Epidemiology and etiology of childhood pneumonia in 2010: estimates of incidence, severe morbidity, mortality, underlying risk factors and causative pathogens for 192 countries

**DOI:** 10.7189/jogh.03.010401

**Published:** 2013-06

**Authors:** Igor Rudan, Katherine L. O’Brien, Harish Nair, Li Liu, Evropi Theodoratou, Shamim Qazi, Ivana Lukšić, Christa L. Fischer Walker, Robert E. Black, Harry Campbell, on behalf of Child Health Epidemiology Reference Group (CHERG)

**Affiliations:** 1 Centre for Population Health Sciences and Global Health Academy, University of Edinburgh Medical School, Edinburgh, Scotland, UK; 2 Department of International Health, Johns Hopkins Bloomberg School of Public Health, Baltimore, MD, USA; 3 Department of Maternal, Newborn, Child and Adolescent Health, World Health Organization, Geneva, Switzerland; 4 Department of Microbiology, Dr Andrija Štampar Institute of Public Health, Zagreb, Croatia

## Abstract

**Background:**

The recent series of reviews conducted within the Global Action Plan for Pneumonia and Diarrhoea (GAPPD) addressed epidemiology of the two deadly diseases at the global and regional level; it also estimated the effectiveness of interventions, barriers to achieving high coverage and the main implications for health policy. The aim of this paper is to provide the estimates of childhood pneumonia at the country level. This should allow national policy–makers and stakeholders to implement proposed policies in the World Health Organization (WHO) and UNICEF member countries.

**Methods:**

We conducted a series of systematic reviews to update previous estimates of the global, regional and national burden of childhood pneumonia incidence, severe morbidity, mortality, risk factors and specific contributions of the most common pathogens: *Streptococcus pneumoniae* (SP), *Haemophilus influenzae* type B (Hib), respiratory syncytial virus (RSV) and influenza virus (flu). We distributed the global and regional–level estimates of the number of cases, severe cases and deaths from childhood pneumonia in 2010–2011 by specific countries using an epidemiological model. The model was based on the prevalence of the five main risk factors for childhood pneumonia within countries (malnutrition, low birth weight, non–exclusive breastfeeding in the first four months, solid fuel use and crowding) and risk effect sizes estimated using meta–analysis.

**Findings:**

The incidence of community–acquired childhood pneumonia in low– and middle–income countries (LMIC) in the year 2010, using World Health Organization's definition, was about 0.22 (interquartile range (IQR) 0.11–0.51) episodes per child–year (e/cy), with 11.5% (IQR 8.0–33.0%) of cases progressing to severe episodes. This is a reduction of nearly 25% over the past decade, which is consistent with observed reductions in the prevalence of risk factors for pneumonia throughout LMIC. At the level of pneumonia incidence, RSV is the most common pathogen, present in about 29% of all episodes, followed by influenza (17%). The contribution of different pathogens varies by pneumonia severity strata, with viral etiologies becoming relatively less important and most deaths in 2010 caused by the main bacterial agents – SP (33%) and Hib (16%), accounting for vaccine use against these two pathogens.

**Conclusions:**

In comparison to 2000, the primary epidemiological evidence contributing to the models of childhood pneumonia burden has improved only slightly; all estimates have wide uncertainty bounds. Still, there is evidence of a decreasing trend for all measures of the burden over the period 2000–2010. The estimates of pneumonia incidence, severe morbidity, mortality and etiology, although each derived from different and independent data, are internally consistent – lending credibility to the new set of estimates. Pneumonia continues to be the leading cause of both morbidity and mortality for young children beyond the neonatal period and requires ongoing strategies and progress to reduce the burden further.

Pneumonia is still the leading cause of child mortality globally [1,2]. However, an increased focus on the reduction of child mortality that arose from the United Nation's Millennium Declaration [3] and the Millennium Development Goal 4 has renewed the interest in developing more accurate estimates of the causes of child deaths. This should inform more effective health policies and track the progress of their impact. In 2001, the Child Health Epidemiology Reference Group (CHERG) – a group of independent technical experts funded by The Gates Foundation and working closely with the World Health Organization (WHO) and UNICEF– set out to systematically review and improve data collection, methods and estimates of the main causes of child deaths for 2000 [4]. Evidence from CHERG estimates – ie, that pneumonia was the leading cause of child mortality –contributed to the initiation of a number of global efforts, such as the Global Action Plan for Pneumonia (GAPP). GAPP was designed to promote the expansion and improvement in community case management, the reduction in risk factors for disease and the support for the massive roll–out of vaccination against *Haemophilus influenzae* type b (Hib) and *Streptococcus pneumoniae* (SP) by countries through support from the GAVI Alliance [5,6]. Those efforts, alongside economic and social developments observed in many low– and middle–income countries over the past decade, have all contributed to a substantial reduction of the burden of morbidity and mortality from childhood pneumonia [7].

CHERG's work also led to several Lancet series that had a substantial impact on global, regional and national–level donors and policy–makers [7–10]. It also inspired similar efforts to address the epidemiology and provide estimates for other causes of the global burden of different diseases [11,12]. The recent series of reviews published in the *Lancet* and *PLoS Medicine*, conducted by CHERG members in collaboration with the WHO, UNICEF and USAID within the Global Action Plan for Pneumonia and Diarrhoea (GAPPD), addressed the epidemiology and the current global burden of the two leading causes of childhood death, pneumonia and diarrhea, in the year 2010–11 [13]. The series also estimated the importance of risk factors [13], effectiveness of interventions [14], barriers to achieving high coverage at the community level [15], validity of coverage measures [16–17] and main implications for health policy [7].

The recent GAPPD reviews focused at the global and regional level [13]. The aim of this paper is to supplement the *Lancet*'s GAPPD series with further information on the underlying models and methods, to augment that already available, and thereby assure that all input data and detailed descriptions of methods are transparently presented and available in an open–access source. Additionally, this paper also provides estimates of childhood pneumonia burden at the country level to allow national policy–makers and other stakeholders to implement the proposed policies in the World Health Organization (WHO) and UNICEF member countries.

## Challenges to estimation of childhood pneumonia burden

**Incidence and severe morbidity.** An accurate estimate of the global, regional and national burden of childhood pneumonia is very difficult to make for a number of reasons. First, the incidence of pneumonia can only be properly assessed through longitudinal community based studies [18]. Such studies are very scarce in low and middle–income countries, where the majority of the pneumonia disease burden occurs, in part because they require a major commitment from both the investigators and research funders in a low–resource setting over an extended period of time. Due to the seasonal nature of pneumonia incidence, which has various peaks in different seasons, studies measuring incidence need to be conducted over full calendar years (or multiple 12–month periods) [19]. The screening of large numbers of children needs to be active, regular and frequent (eg, no longer than 2 weeks between home visits), because recall bias leads to under–estimation especially in large families [19]. In addition to these basic methodological requirements, the most fundamental uncertainty with measuring the incidence of childhood pneumonia in a community setting comes from the choice of case definition and the accuracy of its application by the assessor who establishes the diagnosis. Since pneumonia is actually a diagnosis made on tissue pathology, there is no clinical definition that is fully accurate. In any community–based study on pneumonia incidence, the measured entity is not in fact childhood pneumonia itself, but rather the incidence of children who test positive for the chosen case definition of childhood pneumonia [20]. The case definition is based on a number of symptoms and signs; although the WHO definition of childhood pneumonia (cough or difficulty breathing and an elevated respiratory rate, defined according to the child’s age) is the most frequently used in field studies, other definitions are often encountered in the literature confounding cross study comparisons of incidence. Depending on the combination of sensitivity and specificity of the chosen case definition, the burden of “true” pneumonia in the community of children can be grossly over– or underestimated [20].

A further problem is that the clinical training of assessors differs between the studies and this often affects the application of case definitions, unless the study implementation is highly controlled. Physicians tend to use their own clinical judgment in addition to the case definition. They will be likely to provide more conservative estimates, while community health workers may over–diagnose pneumonia in a community to the level where they consider a high proportion of acute respiratory infections in a child as cases of “pneumonia” [19]. Moreover, it is important to understand for each study whether the investigators attempted to exclude cases of respiratory disease that met the clinical pneumonia case definition but were assessed in some other way as being bronchiolitis, pertussis, measles, or even asthma, malaria or neonatal sepsis.

The effect of these challenges was reflected in the first–ever attempt to estimate the incidence of childhood pneumonia, which identified only 28 studies that met the minimum quality criteria [18]. The incidence of pneumonia reported in these studies still ranged 100–fold between their minimum and maximum reported incidence rates per child–year which could reflect true heterogeneity in the burden of disease or more likely also reflects the challenges of standardizing the epidemiologic study design and its application at the field level [18]. Similar, if not greater problems are encountered with estimating the incidence of severe, life–threatening pneumonia (which requires hospital referral and treatment) in the community. This estimate cannot be based on measures of childhood pneumonia hospitalizations because parents’ care seeking behavior, access to hospitals, and medical professionals’ threshold for admission varies widely within and across geographic settings [19]. There are WHO definitions for severe pneumonia (cough and difficulty breathing with lower chest wall indrawing) and for very severe pneumonia (cough and difficulty breathing with danger signs). These definitions are useful insofar as they are applied at the community level for guiding the case management and referral of children to a hospital, hence are purposefully highly sensitive and poorly specific for truly life threatening disease. Therefore, estimates of the incidence of severe childhood pneumonia in the community are particularly rare. Moreover, great caution must be applied in making comparisons between studies or in combining data across studies to assure that only similarly designed and implemented case definitions are considered together. The best estimates of pneumonia usually come from the control arms of randomized controlled trials of vaccines. This is because severe pneumonia is usually an outcome that is being monitored over a multiple of 12 months, usually with a highly stipulated and rigorously implemented case definition. Such studies provide the best estimates of severe pneumonia in the community that we have today [19].

**Mortality.** Estimating mortality that results from childhood pneumonia in a community also has its significant methodological challenges. Mortality studies require similar study designs to incidence studies, although home visits do not need to be as frequent as in the former, because care–giver recall of a child death is more accurate and long–lasting than of an illness episode [21,22]. Identifying the exact cause of death can be difficult in an appreciable number of cases. The assigned cause of death is usually based on a verbal autopsy provided by a mother or another family member. These are typically based on the report of signs and symptoms around the time of death. Many of them are not specific to pneumonia, but can also be found in children with other conditions, such as sepsis and malaria. In addition, many dying children have suffered from chronic malnutrition and may have other underlying ailments, such as asthma, metabolic disorders, immunodeficient conditions (HIV), sequelae of previous injuries, chronic diarrhea, or congenital defects [23]. They may develop pneumonia in addition to an exacerbation of another ailment, or have concomitant malaria or diarrhoea. In such cases, it is challenging to assign the death of a child to a single cause through verbal autopsy. Furthermore, the clinical signs and symptoms of a pneumonia death overlap with those of other causes of death such as malaria or measles, hence misclassification errors are significant. Moreover, there are studies that focus exclusively on pneumonia as a cause of death, while others are multi–cause mortality studies, documenting the causes of all child deaths in the community. Typically, studies focused exclusively on pneumonia tend to over–estimate its contribution to overall child mortality [24]. This is because in such studies it is more likely that a number of other underlying causes or immediate causes may be misclassified as pneumonia. Therefore, multi–cause mortality studies are preferred as a source of information to single–cause studies [24].

**Risk factors.** In addition to estimating the incidence, severe morbidity and mortality from childhood pneumonia at the global, regional and national level, it is important to understand risk factors that contribute to the development of childhood pneumonia and that may offer clues to prevention of the disease. However, well–conducted studies of pneumonia risk factors in low resource settings are remarkably scarce. There is wide variation among risk factor studies in their focus, study design and outcome: while some explore risk factors associated with incidence of pneumonia at the community level, others focus on the risks that are associated with progression to severe disease in those who already have pneumonia [1]. A third type of study are those that are hospital–based and investigate risk factors associated with progression to death in a child receiving treatment and compare case–fatality rates among different children [1].

Another methodological challenge is that the most commonly investigated risk factors for disease or for death are commonly identified together among cases. For example, undernutrition, use of solid fuels in a household, crowding, lack of exclusive breastfeeding, low degree of maternal education, limited access to secondary care and passive care–seeking behavior are all often characteristics of poor households, where most of the deaths occur. Because of this collinearity, an assessment of the effect size of any particular risk factor in isolation from the role of others will likely lead to gross over–estimation of the true effect size [1,18,19]. Therefore, very large prospective studies are required, based on multivariable study designs, to ensure an adequate number of study participants with heterogeneity in the prevalence of risk factors and thereby allow an accurate assessment of the individual role of each risk factor. Very few such studies exist; this is a permanent research priority, because the effect sizes attributable to individual risk factors in different contexts are still poorly understood [1,18,19].

**Etiological agents.** There is a growing need to identify etiological agents that contribute to the disease development at each of the three levels of severity – episodes of community–acquired pneumonia (incidence), severe pneumonia (severe morbidity) and pneumonia deaths (mortality). This is because vaccines are now available to prevent infections with major pathogens, such and *Streptococcus pneumoniae* (SP), *Haemophilus influenzae type b* (Hib) and *influenza virus* (flu), while a vaccine against respiratory syncytial virus (RSV) is also being actively pursued [25–27]. However, precise estimation of the distribution of the episodes, severe episodes and deaths from childhood pneumonia by etiological agent is even more difficult than estimation of the overall disease burden itself, for a number of reasons. First and foremost, the site of infection – the lung –is generally an inaccessible organ that is in constant contact with the external environment through the naso– and oro–pharynx, which are body sites that are sampling and immunologically responding to potential pathogens. Second, the procedures needed to collect specimens from potential cases are ones that usually require a hospital facility, meaning that studies must be done in places where cases have access to a hospital facility. Such studies also require laboratory facilities that can process samples in a timely fashion and can run a multitude of tests to document presence of pathogens in a child [28]. This means that they tend to be (teaching) hospital–based and therefore do not sample across the whole range of pneumonia cases in a population. Most deaths from pneumonia occur in places where no hospital facility is available, highlighting the nearly inextricable paradox that appropriate studies cannot be done in the places where most of the death burden occurs.

Third, accepting that paradox, even in settings where studies can be done there are further issues. The choice of biological samples (specimen) in which the presence of a potential pathogen should be sought means that multiple body fluids must be collected. Ideally, for a bacterial diagnosis, samples should come from the lung tissue itself (eg, by needle aspirate), from a pleural exudate, or a blood culture sample, but this is often neither feasible nor acceptable in a professional or lay community [28]. As an alternative, analyses of collected sputum or nasopharyngeal swabs can be performed, but their contribution to understanding the etiology is complex since the pathogens identified in these locations are also commonly found among healthy children.

Fourth, the more tests performed, the more agents will be found, and statistical methods to disaggregate and associate individual pathogen contributions to etiology are lacking. This is an increasing problem with modern sensitive techniques like PCR–based tests identifying the presence of often many co–existing and potentially pathogenic agents (whose role in the disease episode is very uncertain). Finally, we don't sufficiently understand the interplay between various pathogens and how a specific time sequence (eg, a viral infection, followed by a bacterial superinfection) may act to compromise the local and/or systemic immune response to cause a serious and life–threatening episode of childhood pneumonia by a pathogen that may otherwise not cause severe disease. Even with sophisticated expansive testing a significant proportion of cases may not have an etiology associated with the case. The meaning of this has to be assessed. Some of these cases may not have pneumonia at all, while other cases may not be associated with an etiology because of statistical methods used, in spite of identification of pathogens in the upper respiratory tract; finally, some may not be assigned to a causal pathogen because of laboratory test insensitivity. There remains therefore a gap in understanding the etiological spectrum of what is clinically defined as pneumonia [25,28].

These complex issues for studying pneumonia etiology are being addressed in a large, 7 country pneumonia etiology study among children (PERCH) [25]. This study is under way and the first results are expected following the completion of the field work (in early 2014) and an analysis period.

Because of the many biologic, epidemiologic, laboratory, and statistical challenges of pneumonia etiology observational studies, the most reliable methods for estimation of the proportional contribution of different pathogens to the burden of childhood pneumonia are vaccine trials [29]. The observed reduction in the incidence of pneumonia (using various case definitions) following vaccination reveals the disease burden attributable to that specific pathogen, once the less than 100% vaccine efficacy of the product is accounted for. This approach also has its limitations, mostly insofar as a vaccine trial can only reveal the burden of one pathogen at a time. For some pathogens (such as SP), not all disease–causing strains may be included in a strain specific vaccine [30]. If the distribution of strains varies by factors that also contribute to variation in pneumonia disease burden (eg, geography, pneumonia case definition, malnutrition, HIV), then careful attention must be paid to applying the vaccine efficacy measures to the appropriate measure of pneumonia disease burden [29]. Also, vaccine–based approach may be very useful in understanding the causal contribution at the level of incidence and severe morbidity, but may be limited in their ability to inform about the pathogen contribution to mortality (which is often a rare event in vaccine trials, where enormous resources are in place that themselves reduce the risk of death). Finally, although it might be ideal to conduct vaccine trials in parallel in many geographic regions using a harmonized protocol to reveal the geographic variability in contribution of pathogens to disease, vaccine trials are not usually designed for the purpose of disease burden estimation; they are also very expensive to conduct, which limits the number of sites where they can be undertaken [31]. They are generally not sufficiently large to have acceptable statistical power to detect a mortality reduction, as there are relatively few deaths in the study population.

Moreover, after a definite proof of vaccine efficacy and effectiveness is established, there are significant ethical issues regarding the conduct of further trials if they necessitate a control arm in which children are not provided what has been shown to be a life–saving vaccine. This self–limits the accumulation of the evidence towards the importance of specific pathogens. An additional layer of complexity comes from the notion that the etiological spectrum may change markedly with increasing severity of disease: at the level of incidence of childhood pneumonia in the community, viral causes seem to be responsible for a majority of episodes. However, a proportion of these cases will result in severe and life–threatening disease. In a sub–sample of severe cases, bacterial agents seem to be over–represented. Evidence from antibiotic treatment trials, from vaccine trials, and from studies of lung puncture studies provide a firm evidence base that episodes of death from pneumonia are dominated by bacterial causes. If true, this would suggest that SP and Hib vaccine probe studies (with “proxy” endpoints of severe episodes prevented) may under–estimate the importance of these agents as a cause of death. Longitudinal studies of mortality in low–income countries that have introduced Hib and SP vaccines recently and that are achieving high vaccine coverage will likely provide confirmatory evidence of that contribution to pneumonia mortality in the coming years [25,26,31].

## An overview of previous estimates

One of the earliest attempts at estimating the global burden of communicable diseases was provided by Cockburn and Assaad in the early 1970s [32]. Bulla and Hitze built on their work by specifically addressing the contribution of acute respiratory infections [33]. Almost a decade later, Leowski [34] used data from 39 countries to estimate that acute respiratory infections may have been causing about 4 million child deaths each year: 2.6 million in infants and further 1.4 million in children aged 1–4 years. In the early 1990s Garenne et al. [35] further refined these estimates using an epidemiological model that explored the association between all–cause child mortality and the proportion of deaths attributable to acute respiratory infections, showing that between 20–33% of child deaths were associated with respiratory infections [35,36].

The 21^st^ century has seen a much larger number of efforts, mainly designed and led by CHERG and their partners, which further improved the understanding of the epidemiology and etiology of childhood pneumonia. The first estimate of global incidence of childhood pneumonia was provided by Rudan et al. [18] for 2000. In parallel, a refined estimate of childhood pneumonia mortality for the same year, based mainly on single–cause studies, was provided by Williams et al. [37]. The first estimate of pneumonia mortality from multi–cause studies was published by Black et al. in CHERG's paper on the causes of global child mortality in the year 2000 [4]. Then, estimates underwent further refinements and updates. An updated estimate of childhood pneumonia mortality for 2008 in post–neonatal children in low and middle–income countries, based on single–cause studies, was provided by Theodoratou et al. [38]. Estimates based on multi–cause studies underwent three updates: for the period 2000–2003 by Bryce et al. [39]; for 2008 by Black et al. [40]; and for 2010 by Li et al. [41].

The first comprehensive assessment of the burden of severe pneumonia according to the WHO's definition and the role of risk factors was provided by Rudan et al. [1,18]. This work was followed by the first attempt to estimate the global burden of childhood pneumonia on health systems; Nair et al. [42] used both published and unpublished information to calculate the number of hospitalizations for severe pneumonia, a number which is smaller than the estimate of cases of severe pneumonia in the community because of lack of access and/or care–seeking in many settings.

Once the “envelopes” for the burden of pneumonia incidence, severe morbidity and mortality from pneumonia in 2000 were provided, a series of efforts attempted to estimate the proportion of the burden at each level of severity that can be attributed to the main etiological agents that cause pneumonia. O'Brien et al. [43] developed the first global, regional and country estimates for the morbidity and mortality from *Streptococcus pneumoniae*, Watt et al. for *Haemophilus influenzae* type b [44], while Nair et al. generated global and regional estimates for RSV [45] and for influenza [46].

The estimates of pneumonia incidence, severe pneumonia cases, severe pneumonia hospitalizations, pneumonia mortality, and cause specific estimates are based on different and almost entirely independent sources of information, which allows for assessments of validity and consistency between the various estimates. Validation of these estimates can be approached in various ways. A few examples include: (i) an assessment of the measured proportion of all pneumonia cases that are categorized as severe; (ii) the ratio between the estimates of severe episodes and deaths, and also (iii) between all pneumonia episodes and deaths. These proportions and ratios need to largely support the observed case–fatality rates typically seen in both community–based and hospital–based data sets from individual studies. Moreover, the sum of etiology specific fractions attributed to different pathogens needs to fit within the overall burden of incidence, severe morbidity and mortality. For the Hib and pneumococcal pathogen specific estimates, they must fit within these envelopes by definition, since the methodology to estimate the absolute burden was a proportional approach – but this was not the approach for the estimation of the RSV or influenza burden. The ratios between different pathogens were also found to broadly reflect those observed in the high quality field studies or hospital–based studies further validating the estimates. Towards the end of the past decade it was notable that, regardless of all methodological challenges and uncertainty inherent to this research, all the major estimates from different sources were increasingly consistent with each other and provided a clearer global and regional picture of the burden of childhood pneumonia and its causing pathogens, albeit with wide uncertainty bounds around the point estimates [40–46]. This paper therefore brings all the estimates together and provides an update for 2010–11, in which all information is provided in a single analysis, and where country–level estimates are also be provided.

## METHODS

Many steps are required to develop an internally consistent estimate of global, regional and national burden of childhood pneumonia based on best available evidence. To fully explain our approach, we developed a table (Online Supplementary Document(Online Supplementary Document)) which all input data, assumptions, methods, solutions to specific problems or dilemmas, formulae for calculation of different parameters, and the interim and final estimates are provided. In this section, we present a summary for those steps, list all sources of data and explain the rationale for each subsequent step.

## Input data for country–level populations and prevalence of risk factors for pneumonia incidence

Initially, we list 192 countries by World Health Organization's regional classification, with 6 main regions (the Americas (AMRO), Africa (AFRO), Eastern Mediterranean region (EMRO), European region (EURO), Western Pacific region (WPRO) and South–East Asian region (SEARO)) and further divisions by the level of development into “A”, “B”, “C”, “D” and “E” sub–regions [47]. For each country, an estimate of the population of children under the age of 5 years in 2010 was obtained from the UN's Population Division [48]. Then, the 5 most important risk factors for childhood pneumonia incidence were identified. They were selected based on consistently significant effects in multivariate study designs and previous meta–analyses [1,18]. They are: malnutrition (weight–for–age z<–2), low birth weight (≤2500 g), non–exclusive breastfeeding (in the first 4 months), solid fuel use (“yes”) and crowding (7 or more persons sharing the same household) [1,18]. The data on the prevalence of exposure to each of those 5 risk factors in each country in the year 2010(or the closest year with available data) was obtained from the recent Demographic and Health Surveys (DHS) and Multiple Indicator Cluster Surveys (MICS) [49,50]. For all countries in which data on the prevalence of exposure were not available, the prevalence was imputed based on the regional mean value, which was weighted by population size of all countries with any data. The effect size of each risk factor on pneumonia incidence was assessed through meta–analysis of the studies that reported multivariable analyses of risk factor's odds ratios (OR) in low and middle–income countries. The meta–estimates of odds ratios assigned to each risk factor were: 1.8 for malnutrition, 1.4 for low birth weight, 1.3 for non–exclusive breastfeeding, 1.8 for use of solid fuels and 2.0 for crowding. In high–income countries, where less than 2% of all cases of community–acquired pneumonia occur, we did not use the model based on risk factors but rather applied “flat” rates of incidence for “A”, “B” and “C” regions based on several high–quality studies (see Online Supplementary Document(Online Supplementary Document)), and which ranged between 0.015 and 0.060 episodes/child–year (see later). For the proportion of severe episodes in each high–income region we used one single rate which was the median of all available studies (26.7%, see later).

## Computation of country–level incidence of pneumonia and severe pneumonia

In all LMIC countries, we multiplied the number of children in each country by the prevalence of exposure to each of the 5 risk factors. This provided an estimate of the absolute number of exposed children in each country who were at excess risk of developing childhood pneumonia in the year 2010. We then calculated the proportion (ie, a weighted mean) of all children in each LMIC region and country exposed to each of the 5 risk factors; then, in each country, we multiplied the proportion of children who were above, or below, the regional exposure level with the meta–estimate of the odds ratio attributable to each of the 5 risk factors.

The number of pneumonia cases in each low and middle–income country (LMIC) was calculated using a model based on the epidemiological concept of potential impact fraction [51], as follows:


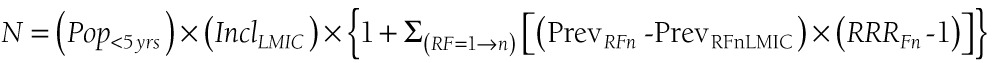


where N is the number of new episodes of childhood pneumonia per year in each country, Pop_<5yrs_is the population of children aged 0–4 years in each LMIC, Inc_LMIC_ is the estimated incidence of clinical pneumonia for all LMIC, Prev_RFn_ is the prevalence of exposure to *n*–th risk factor among those under 5-year in the country of interest, Prev_RFnLMIC_ is the prevalence of exposure to *n*^th^ risk factor among under–fives in all LMIC, and RR_RFn_ is the relative risk for developing clinical pneumonia associated with the *n*^th^ risk factor (see Online Supplementary Document(Online Supplementary Document) for further details).

The incidence of pneumonia for all LMIC was derived from35 community–based studies published between1990 and 2012 (references shown in Online Supplementary Document(Online Supplementary Document)), by using the median value (0.22 episodes/child–year) and inter–quartile range (IQR) 0.11–0.51 as confidence intervals.

Although there are many possible methods to distribute the global and/or regional burden estimate among individual countries, the approach used above is our preferred solution because it is epidemiologically sound and biologically intuitive insofar as it is based on the country specific prevalence of known risk factors for pneumonia, and because it can be explained in a transparent and accessible manner. Although more complex models exist, our experience is that these sometimes result in implausibly high or low estimates for some countries, the cause of which is difficult to disentangle. This model, because of its computational simplicity and epidemiologic basis, has not suffered from this problem. The model has also been shown to distribute a known overall burden by specific countries in the absence of truly nationally representative information from many (or, in this case, from most) countries in a way which is consistent with clinical and epidemiologic knowledge.

The proportion of cases of severe pneumonia (based on the WHO definition that requires presentation of lower chest wall indrawing, and represents an indication for hospitalization) for LMIC was computed based on 9 community–based studies in LMIC that reported the proportion of severe pneumonia episodes among all pneumonia episodes (references shown in Online Supplementary Document(Online Supplementary Document)). The median value was 11.5% (IQR 8.0–33.0%). The incidence of pneumonia in high–income countries, based on a smaller number of very large, high–quality studies (references shown in Online Supplementary Document(Online Supplementary Document)), was also estimated using medians (and IQR): it was 0.015 e/cy in EUROA and AMROA regions; 0.030 e/cy in EURO Band 0.060 in EURO C. The mean of those values (for the whole HIC region), weighted by their under–five population size, was0.024 e/cy [52]. Approximately 26.7% (IQR 20.0–46.7%) of those episodes are estimated to progress to severe pneumonia, based on several studies from high–income countries (references shown in Online Supplementary Document(Online Supplementary Document)). The estimates for the number of incident and severe pneumonia episodes derived in this way did not account for the use and effect of pneumococcal conjugate vaccine (PCV) and Hib vaccination coverage in 2010 at this stage of the estimation process, so the values from this step are not considered the final pneumonia burden numbers.

### Etiologic fractions of pneumonia and severe pneumonia cases

We split both the incidence and severe morbidity of childhood pneumonia by etiological agents while adjusting for the effects PCV and Hib vaccines according to country specific coverage values provided for 2010 by the UNICEF [53]. In doing so, we used the proportional contributions to all childhood pneumonia and severe childhood pneumonia from previous burden estimates on SP [43], Hib [44], RSV [45] and influenza [46] and accounted for vaccine efficacy and serotype distribution of pneumococcal disease as well as dual use of Hib and PCV where relevant. All further details are available in Online Supplementary Document(Online Supplementary Document).

### Country–specific estimates of the number of deaths from childhood pneumonia

This was available for 2010 from Li et al. [41]. A more recent update was made available by the UN Inter–Agency Group for Child Mortality Estimation IGME in UNICEF's 2012 report, which we term a “2010–2011” estimate [54]. Given the important focus on child mortality, and relatively minor differences compared with the Li 2010 estimates, we elected to use the 2010–11estimates for the envelopes of pneumonia deaths by country. The same decision was made in the Lancet's series [13]. The only methodological problem with this decision is a separation of Sudan and introduction of the new country – South Sudan from 2011, but we presented our results on mortality for both Sudan nations combined, and kept it within the EMRO region, although South Sudan belongs to AFRO region in the new classification [47].

### Proportional split of pneumonia deaths by etiological agent

To estimate the fraction of pneumonia deaths attributable to SP and Hib, we used the meta–analysis of the efficacy of PCV and Hib vaccines against chest X–ray confirmed pneumonia as has been described earlier (43,44), based on the assumption that the etiologic fraction of these bacteria among these particular cases approximates the etiologic fraction among the deaths. The values (33.0% for SP and 21.3% for Hib) were then adjusted by country for the use of PCV and Hib vaccine to derive the final SP and Hib proportions [43,44,53]. Since the global disease burden estimates for flu and RSV pneumonia were not able to give point estimates and confidence intervals due to lack of data we did not attempt to go beyond the published global and regional estimates for these conditions and so did not attempt to derive national–level estimates [45,46].

## RESULTS

**Table 1** presents our estimates for 192 countries, grouped by the WHO regions: Africa (AFRO), the Americas (AMRO), Eastern Mediterranean region (EMRO), South–East Asian region (SEARO), Western Pacific region (WPRO) and European region (EURO). Several main results emerge from the presented figures. First, the population of under–five children in the world increased from 604.9 million to 633.5 million between 2000 and 2010, but the majority of the increase was observed in low– and middle–income countries (523.3 to 547.3 million), and only a smaller share in high–income countries (81.6 to 86.2 million). Holding all else constant, an increase in total child population would increase the absolute number of pneumonia cases; however, the number of cases has decreased over the past decade, because the incidence has decreased substantially. When presenting our estimates of incidence for 2000, we reported on 28 studies published between 1960 and 2000 that suggested an estimated incidence of 0.29 (0.21–0.71) episodes per child–year globally [18]. In this most recent estimate, we used 35 studies published between 1990 and 2010 with a median incidence of 0.22 (0.11–0.51). This is a notable reduction, of nearly 25%, over a period of a decade. In high–income countries we gathered more data over the past decade, and a very rough estimate of 0.05 e/cy, based on two very large, but historic studies in the USA and the UK [55,56], was refined and replaced with the data from 9 more contemporary studies, which provide a community–based incidence of 0.015 e/cy (0.012–0.020) for HIC only (WHO's “A” regions), a more plausible estimate for the modern industrial societies.

**Table 1:** Estimates of the number of new episodes (incidence) of community–acquired pneumonia in 2010 in children 0–4 years of age in 192 countries, shown as national–level totals (incidence, all ALRI) and by causative pathogens (SP, Hib, RSV and flu); estimates of the number of new severe episodes (according to WHO's definition) in the year 2010 that require hospitalizations, shown as national–level totals (severe episodes, all ALRI) and by causative pathogens (SP, Hib, RSV and flu); and estimates of the number of child deaths attributable to pneumonia in 2011 (mortality, all ALRI) and the proportion of deaths caused by SP and Hib

			New episodes (incidence)					New severe episodes (severe morbidity)					Deaths (mortality)			
**Country**	**WHO Region**	**Population 0–4 years**	**All ALRI**	**SP**	**Hib**	**RSV**	**FLU**	**All ALRI**	**SP**	**Hib**	**RSV**	**FLU**	**All ALRI**	**SP**	**Hib**	RSV, FLU*
**AFRO REGION**																
Algeria	AfroD	3446548	470713	34251	4697	135754	80351	53790	10297	783	7315	2251	2440	804	148	N/A
Angola	AfroD	3377576	856794	62241	9674	247099	146255	97936	18712	1613	13293	4090	20429	6733	1398	N/A
Benin	AfroD	1506408	424074	30705	5895	122303	72389	48501	9231	983	6558	2018	6281	2070	522	N/A
Burkina Faso	AfroD	2955148	1047365	76085	11826	302060	178785	119719	22874	1972	16250	5000	17933	5911	1227	N/A
Cameroon	AfroD	3054802	790160	56858	14815	227882	134880	90462	17094	2470	12143	3736	13341	4397	1463	N/A
Cape Verde	AfroD	50634	9874	691	395	2848	1686	1136	208	66	148	45	39	13	8	N/A
Chad	AfroD	2006165	678297	48155	19812	195621	115785	77827	14477	3304	10285	3164	14683	4840	2390	N/A
Comoros	AfroD	122296	38380	2769	645	11069	6552	4392	832	108	591	182	377	124	37	N/A
Equ. Guinea	AfroD	107207	16341	1144	654	4713	2789	1879	344	109	244	75	402	132	85	N/A
Gabon	AfroD	185179	36186	2579	943	10436	6177	4149	775	157	551	170	291	96	43	N/A
Gambia	AfroD	287078	79805	2667	802	23016	13623	8746	802	134	1338	412	987	171	56	N/A
Ghana	AfroD	3532887	795448	57857	8199	229407	135783	90905	17394	1367	12357	3802	7808	2573	490	N/A
Guinea	AfroD	1657883	546525	39262	10948	157618	93292	62586	11804	1826	8385	2580	7689	2534	895	N/A
Guin.–Bissau	AfroD	240350	75199	5429	1216	21687	12836	8605	1632	203	1159	357	1592	525	152	N/A
Liberia	AfroD	680701	212990	15195	5418	61426	36357	24419	4568	903	3245	999	1611	531	232	N/A
Madagascar	AfroD	3305278	1051407	76189	13932	303226	179475	120231	22906	2323	16272	5007	8004	2638	637	N/A
Mali	AfroD	2911668	932894	67350	15086	269047	159245	106745	20248	2516	14384	4426	23947	7893	2292	N/A
Mauritania	AfroD	513267	144982	10415	2904	41813	24748	16603	3131	484	2224	684	2099	692	244	N/A
Mauritius	AfroD	84433	13518	985	117	3899	2307	1544	296	20	210	65	20	7	1	N/A
Niger	AfroD	3084517	1127652	81210	20418	325215	192490	129082	24415	3405	17344	5337	19004	6264	2018	N/A
Nigeria	AfroD	26568927	7339761	513783	293590	2116787	1252897	844072	154465	48956	109729	33763	121201	39948	25767	N/A
S. Tome & P'e	AfroD	23490	5118	373	46	1476	874	585	112	8	80	25	79	26	4	N/A
Senegal	AfroD	2081483	591373	42853	7836	170552	100947	67625	12883	1307	9152	2816	4612	1520	367	N/A
Seychelles	AfroD	5623	862	63	7	248	147	98	19	1	13	4	2	1	0	N/A
Sierra Leone	AfroD	969597	315676	22866	4286	91041	53886	36101	6874	715	4883	1503	7262	2393	591	N/A
Togo	AfroD	862745	280487	20292	4082	80893	47879	32083	6101	681	4334	1333	3321	1095	288	N/A
Zimbabwe	AfroD	1692247	349031	25271	4852	100661	59580	39918	7598	809	5397	1661	2461	811	205	N/A
Botswana	AfroE	225120	47818	3347	1913	13791	8162	5499	1006	319	715	220	159	52	34	N/A
Burundi	AfroE	1184632	349477	25440	3373	100789	59656	39933	7648	562	5433	1672	7259	2393	428	N/A
Cen. Afr. Rep.	AfroE	651222	195417	13981	4538	56358	33358	22394	4203	757	2986	919	3911	1289	520	N/A
Congo	AfroE	623244	168619	12244	1959	48630	28783	19275	3681	327	2615	805	2001	659	141	N/A
Cote d'Ivoire	AfroE	2969425	985611	71421	13060	284250	168244	112707	21472	2178	15253	4693	11003	3626	875	N/A
D. Rep. Congo	AfroE	11848026	3671614	263117	80589	1058894	626745	420631	79104	13438	56194	17291	86897	28641	10986	N/A
Eritrea	AfroE	861496	208035	15163	1802	59997	35512	23766	4559	301	3238	996	2419	797	129	N/A
Ethiopia	AfroE	11931668	3367561	240540	82471	971205	574843	386005	72317	13752	51372	15807	37269	12284	5196	N/A
Kenya	AfroE	6664323	1645189	119118	22871	474473	280834	188157	35812	3814	25440	7828	17064	5624	1419	N/A
Lesotho	AfroE	274307	58335	4224	811	16824	9958	6672	1270	135	902	278	607	200	50	N/A
Malawi	AfroE	2714859	658512	47877	7004	189915	112408	75261	14394	1168	10225	3146	6932	2285	448	N/A
Mozambique	AfroE	3876419	1155781	83373	19438	333327	197292	132266	25065	3241	17806	5479	13167	4340	1307	N/A
Namibia	AfroE	286374	63796	4619	887	18399	10890	7296	1389	148	987	304	287	95	24	N/A
Rwanda	AfroE	1830654	397910	13638	3991	114757	67923	43646	4100	666	6659	2049	4145	734	236	N/A
South Africa	AfroE	5041132	705554	33436	14342	203482	120438	78749	10052	2392	11357	3494	5156	1218	583	N/A
Swaziland	AfroE	156715	28802	2091	344	8306	4916	3293	629	57	446	137	471	155	34	N/A
Uganda	AfroE	6465275	1745727	126241	25969	503468	297996	199697	37953	4330	26961	8296	21181	6981	1876	N/A
U. R. Tanzania	AfroE	8009544	2151379	156285	24291	620458	367240	245913	46986	4051	33378	10270	17467	5757	1195	N/A
Zambia	AfroE	2412190	576056	41709	8008	166135	98333	65882	12539	1335	8908	2741	6141	2024	511	N/A
**AMRO REGION**																
Canada	AmroA	1884546	25275	866	271	13709	8032	6438	604	105	3774	755	27	5	2	N/A
Cuba	AmroA	569056	8208	598	79	4452	2609	2178	417	31	1140	228	63	21	4	N/A
USA	AmroA	21650217	313322	22733	3845	169946	99574	83169	15868	1489	43355	8671	799	263	59	N/A
Antigua & B'a	AmroB	7756	686	50	6	198	117	78	15	1	41	8	0	0	0	N/A
Argentina	AmroB	3385831	311588	22663	3212	89862	53188	35609	6814	536	18616	3723	952	314	60	N/A
Bahamas	AmroB	25507	2514	182	23	725	429	287	55	4	151	30	25	8	1	N/A
Barbados	AmroB	14562	1377	60	19	397	235	153	18	3	87	17	4	1	0	N/A
Belize	AmroB	36599	4795	349	46	1383	819	548	105	8	287	57	9	3	1	N/A
Brazil	AmroB	15156449	1497706	95518	14711	431938	255658	169535	28717	2453	91150	18230	3079	916	181	N/A
Chile	AmroB	1219437	88722	6448	973	25588	15145	10141	1938	162	5296	1059	145	48	10	N/A
Colombia	AmroB	4497661	488486	31421	6092	140879	83385	55372	9446	1016	29585	5917	1530	459	113	N/A
Costa Rica	AmroB	362979	37185	1272	425	10724	6348	4080	382	71	2389	478	24	4	2	N/A
Dominica	AmroB	5924	703	51	6	203	120	80	15	1	42	8	0	0	0	N/A
Dominican R.	AmroB	1054063	121820	8813	1773	35133	20795	13934	2650	296	7239	1448	587	193	51	N/A
El Salvador	AmroB	616802	72388	3616	829	20877	12357	8079	1087	138	4515	903	221	54	15	N/A
Grenada	AmroB	9687	1021	74	10	295	174	117	22	2	61	12	0	0	0	N/A
Guyana	AmroB	64818	7186	523	72	2072	1227	821	157	12	429	86	19	6	1	N/A
Honduras	AmroB	966002	184407	13435	1658	53183	31478	21068	4039	277	11036	2207	478	157	26	N/A
Jamaica	AmroB	246543	31065	2264	269	8959	5303	3549	681	45	1860	372	104	34	6	N/A
Mexico	AmroB	11094854	1110027	40375	11872	320132	189482	122060	12138	1980	71108	14222	4069	759	248	N/A
Panama	AmroB	345142	38834	2112	415	11200	6629	4354	635	69	2404	481	128	33	8	N/A
Paraguay	AmroB	740282	139661	10141	1623	40278	23840	15965	3049	271	8330	1666	368	121	26	N/A
St. Kitts & N's	AmroB	4582	441	32	4	127	75	50	10	1	26	5	0	0	0	N/A
Saint Lucia	AmroB	15115	1492	109	14	430	255	170	33	2	89	18	0	0	0	N/A
St. Vinc. & G's	AmroB	9254	967	70	8	279	165	110	21	1	58	12	1	0	0	N/A
Suriname	AmroB	47543	6578	477	85	1897	1123	752	143	14	392	78	20	7	2	N/A
Trinidad & Tobago	AmroB	95484	9784	710	114	2822	1670	1118	214	19	584	117	38	13	3	N/A
Uruguay	AmroB	246446	17570	647	188	5067	2999	1933	194	31	1125	225	53	10	3	N/A
Venezuela	AmroB	2926202	308502	22291	4789	88972	52661	35295	6702	799	18310	3662	927	305	85	N/A
Bolivia	AmroD	1234922	137114	9915	2040	39544	23405	15685	2981	340	8145	1629	1909	629	169	N/A
Ecuador	AmroD	1469919	163860	10901	1437	47257	27971	18596	3277	240	9934	1987	712	219	38	N/A
Guatemala	AmroD	2167408	481781	35042	4966	138946	82240	55058	10535	828	28785	5757	2012	663	126	N/A
Haiti	AmroD	1237203	345081	24156	13803	99521	58905	39684	7262	2302	19842	3968	4090	1348	870	N/A
Nicaragua	AmroD	677569	141434	10304	1272	40790	24143	16159	3098	212	8464	1693	503	166	28	N/A
Peru	AmroD	2909336	313170	12566	3545	90318	53458	34584	3778	591	19904	3981	1040	211	67	N/A
**EMRO REGION**																
Bahrain	EmroB	93006	9763	327	91	2816	1667	1070	98	15	227	101	5	1	0	N/A
Cyprus	EmroB	63553	7253	528	70	2092	1238	829	159	12	156	69	1	0	0	N/A
Iran (Isl. Rep.)	EmroB	6149331	729564	51069	29183	210406	124537	83900	15354	4866	15102	6712	4168	1374	886	N/A
Jordan	EmroB	816013	87843	6400	790	25334	14995	10036	1924	132	1893	841	268	88	15	N/A
Kuwait	EmroB	281414	29357	994	284	8467	5011	3218	299	47	681	303	38	7	2	N/A
Lebanon	EmroB	321684	35518	2569	517	10243	6063	4063	773	86	760	338	49	16	4	N/A
Libyan A. J.	EmroB	715540	80748	5883	726	23288	13784	9225	1769	121	1740	773	60	20	3	N/A
Oman	EmroB	281883	32111	1074	300	9261	5481	3518	323	50	746	332	25	4	1	N/A
Qatar	EmroB	90524	9669	331	97	2788	1650	1061	100	16	224	100	4	1	0	N/A
Saudi Arabia	EmroB	3145187	337985	11445	3273	97475	57694	37052	3441	546	7842	3485	372	65	20	N/A
Syrian A. R.	EmroB	2493561	280849	20309	4178	80997	47941	32127	6106	697	6006	2669	572	189	51	N/A
Tunisia	EmroB	868231	99837	6989	3993	28793	17042	11481	2101	666	2067	919	209	69	44	N/A
U. A. Emir.	EmroB	420630	46752	1660	517	13483	7981	5137	499	86	1079	480	14	3	1	N/A
Afghanistan	EmroD	5545968	2040302	146694	39565	588423	348280	233617	44102	6598	43379	19280	30913	10189	3494	N/A
Djibouti	EmroD	113169	24926	1808	306	7189	4255	2850	544	51	535	238	446	147	33	N/A
Egypt	EmroD	9008118	680363	47625	27215	196217	116138	78242	14318	4538	14084	6259	4765	1570	1013	N/A
Iraq	EmroD	5188175	893131	62519	35725	257579	152457	102710	18796	5957	18488	8217	7568	2494	1609	N/A
Morocco	EmroD	3021924	385554	27959	3343	111194	65814	44029	8406	557	8316	3696	3103	1019	165	N/A
Pakistan	EmroD	21418111	6728235	487755	86960	1940423	1148510	769337	146640	14501	144236	64105	64853	21376	5039	N/A
Somalia	EmroD	1667479	650669	45547	26027	187653	111069	74827	13693	4340	13469	5986	18089	5962	3846	N/A
Sudan	EmroD	6391368	2061300	148754	34001	594479	351864	235876	44722	5670	43989	19550	26894	8864	3681	N/A
Yemen	EmroD	4057096	1150463	83436	14494	331793	196384	131540	25084	2417	24673	10966	15193	5008	1152	N/A
**SEARO REGION**																
Indonesia	SearoB	21578876	3918360	274285	156734	1130055	668864	450611	82462	26135	99135	22531	19147	6311	4071	N/A
Sri Lanka	SearoB	1892699	433688	31610	3757	125076	74030	49545	9503	626	11425	2597	298	98	16	N/A
Thailand	SearoB	4360687	648021	45361	25921	186889	110617	74522	13638	4322	16395	3726	903	298	192	N/A
Timor Leste	SearoB	192839	67370	4716	2695	19429	11500	7748	1418	449	1704	387	489	161	104	N/A
Bangladesh	SearoD	14707333	4484527	326317	44752	1293338	765509	512461	98105	7462	117940	26805	18310	6035	1114	N/A
Bhutan	SearoD	70891	12773	894	511	3684	2180	1469	269	85	323	73	152	50	32	N/A
DPR of Korea	SearoD	1704446	393494	27545	15740	113484	67169	45252	8281	2625	9955	2263	1744	575	371	N/A
India	SearoD	127960004	35361230	2475286	1414449	10198179	6036162	4066541	744177	235859	894639	203327	388144	127932	82519	N/A
Maldives	SearoD	25984	4061	284	162	1171	693	467	85	27	103	23	6	2	1	N/A
Myanmar	SearoD	3956305	1213300	84931	48532	349916	207110	139530	25534	8093	30697	6976	9129	3009	1941	N/A
Nepal	SearoD	3506023	832451	58272	33298	240079	142099	95732	17519	5552	21061	4787	5501	1813	1170	N/A
**WPRO REGION**																
Australia	WproA	1457527	32776	1204	385	17778	10416	8374	841	149	2724	1654	38	7	3	N/A
Brunei D'lam	WproA	37385	899	65	9	488	286	239	46	3	70	42	3	1	0	N/A
Japan	WproA	5430793	135770	9504	5431	73642	43148	36251	6634	2103	10150	6163	231	76	49	N/A
New Zealand	WproA	311974	7036	264	90	3816	2236	1800	184	35	583	354	31	6	2	N/A
Singapore	WproA	230550	5764	403	231	3126	1832	1539	282	89	431	262	9	3	2	N/A
Cambodia	WproB	1491690	373583	27150	4096	107741	63771	42699	8162	683	12489	7583	2101	693	140	N/A
China	WproB	81595595	6488544	454198	259542	1871296	1107594	746183	136551	43279	208931	126851	43089	14202	9161	N/A
Cook Islands	WproB	2096	210	15	2	61	36	24	5	0	7	4	0	0	0	N/A
Fiji	WproB	89552	14426	1051	125	4161	2463	1648	316	21	484	294	30	10	2	N/A
Kiribati	WproB	9948	1625	118	18	469	277	186	35	3	54	33	19	6	1	N/A
Lao Peop's DR	WproB	682861	212441	15325	3573	61268	36264	24312	4607	596	7049	4280	1076	355	107	N/A
Malaysia	WproB	2828151	285716	20781	2945	82400	48772	32652	6248	491	9559	5804	199	66	12	N/A
Marshall Isl.	WproB	5400	934	59	10	269	159	106	18	2	32	19	5	2	0	N/A
Micronesia	WproB	13237	2620	118	50	756	447	292	35	8	91	55	23	5	2	N/A
Mongolia	WproB	296799	60292	4389	582	17388	10292	6889	1320	97	2019	1226	332	109	20	N/A
Nauru	WproB	1025	97	7	1	28	16	11	2	0	3	2	1	0	0	N/A
Niue	WproB	152	15	1	0	4	3	2	0	0	1	0	0	0	0	N/A
Palau	WproB	2046	211	12	4	61	36	24	3	1	7	4	0	0	0	N/A
Papua N. G.	WproB	962437	166267	11905	3755	47951	28382	19051	3579	626	5476	3325	2038	672	264	N/A
Philippines	WproB	11254421	2428448	170059	96399	700364	414536	279254	51127	16075	78227	47495	8974	2958	1896	N/A
R. of Korea	WproB	2371820	249811	17487	9992	72045	42643	28728	5257	1666	8044	4884	56	18	12	N/A
Samoa	WproB	22338	3377	245	43	974	576	386	74	7	113	68	7	2	1	N/A
Solomon Isl.	WproB	79962	19101	1381	290	5509	3261	2185	415	48	635	386	59	20	5	N/A
Tonga	WproB	13792	2223	162	19	641	379	254	49	3	75	45	4	1	0	N/A
Tuvalu	WproB	1015	126	9	2	36	22	14	3	0	4	3	0	0	0	N/A
Vanuatu	WproB	33152	8344	584	334	2406	1424	960	176	56	269	163	9	3	2	N/A
Viet Nam	WproB	7185862	1728193	124101	35174	498411	295003	197920	37310	5865	57086	34660	3553	1171	420	N/A
**EURO REGION**																
Andorra	EuroA	4001	58	4	1	31	18	15	3	0	9	2	0	0	0	N/A
Austria	EuroA	386431	5604	406	78	3040	1781	1488	283	30	913	186	5	2	0	N/A
Belgium	EuroA	616259	8882	647	80	4817	2823	2356	452	31	1456	296	7	2	0	N/A
Croatia	EuroA	389100	5610	409	52	3043	1783	1488	285	20	919	187	8	2	0	N/A
Czech Rep.	EuroA	547804	7892	575	68	4280	2508	2093	401	26	1294	263	23	7	1	N/A
Denmark	EuroA	326007	4413	168	55	2394	1402	1129	117	21	770	157	5	1	0	N/A
Estonia	EuroA	78229	1129	82	12	613	359	300	57	5	185	38	2	1	0	N/A
Finland	EuroA	299477	4314	314	37	2340	1371	1144	219	14	708	144	7	2	0	N/A
France	EuroA	3974436	53589	2019	534	29067	17031	13698	1409	207	9391	1910	61	12	3	N/A
Germany	EuroA	3466740	49718	3450	516	26967	15800	13146	2408	200	8192	1666	67	21	4	N/A
Greece	EuroA	586137	8500	615	118	4610	2701	2257	430	46	1385	282	35	12	3	N/A
Hungary	EuroA	490804	7071	515	61	3835	2247	1875	360	24	1160	236	27	9	1	N/A
Iceland	EuroA	23511	339	25	3	184	108	90	17	1	56	11	0	0	0	N/A
Ireland	EuroA	358318	5011	282	53	2718	1592	1307	197	21	847	172	4	1	0	N/A
Israel	EuroA	735243	10618	772	113	5759	3375	2818	539	44	1737	353	13	4	1	N/A
Italy	EuroA	2901653	41871	3047	418	22711	13307	11109	2127	162	6856	1395	30	10	2	N/A
Luxembourg	EuroA	28783	389	15	4	211	124	100	11	1	68	14	0	0	0	N/A
Malta	EuroA	19130	278	20	4	151	88	74	14	2	45	9	0	0	0	N/A
Monaco	EuroA	2001	29	2	0	16	9	8	1	0	5	1	0	0	0	N/A
Netherlands	EuroA	934218	12528	435	126	6795	3981	3192	303	49	2208	449	18	3	1	N/A
Norway	EuroA	303047	4085	150	45	2216	1298	1044	105	17	716	146	3	1	0	N/A
Poland	EuroA	1933388	27852	2030	241	15107	8851	7388	1417	93	4568	929	126	41	7	N/A
Portugal	EuroA	516604	7448	542	69	4040	2367	1976	379	27	1221	248	3	1	0	N/A
San Marino	EuroA	1401	20	1	0	11	6	5	1	0	3	1	0	0	0	N/A
Serbia & Montenegro	EuroA	604144	8747	634	110	4744	2780	2322	443	43	1428	290	30	10	2	N/A
Slovakia	EuroA	275895	3688	123	34	2000	1172	938	86	13	652	133	35	6	2	N/A
Slovenia	EuroA	99368	1433	104	14	777	455	380	73	5	235	48	2	1	0	N/A
Spain	EuroA	2521375	36353	2647	339	19718	11553	9644	1848	131	5958	1212	50	16	3	N/A
Sweden	EuroA	557426	7682	382	72	4167	2441	1989	266	28	1317	268	10	2	1	N/A
Switzerland	EuroA	376228	5431	395	56	2946	1726	1441	276	22	889	181	3	1	0	N/A
UK	EuroA	3765820	50844	1913	560	27578	16158	13000	1335	217	8898	1810	165	32	10	N/A
Albania	EuroB	207681	6230	436	249	3379	1980	1664	304	96	981	200	66	22	14	N/A
Bosnia & Herzegovina	EuroB	164958	4784	346	67	2595	1520	1270	242	26	780	159	24	8	2	N/A
Bulgaria	EuroB	373095	10245	470	122	5557	3256	2643	328	47	1763	359	219	50	15	N/A
Georgia	EuroB	256459	7488	539	143	4061	2380	1990	376	55	1212	247	108	36	12	N/A
Romania	EuroB	1079244	32377	2266	1295	17561	10290	8645	1582	501	5100	1037	807	266	172	N/A
FYR Macedonia	EuroB	111863	3236	235	39	1755	1029	859	164	15	529	108	10	3	1	N/A
Turkey	EuroB	6412702	172393	6203	1724	93506	54786	43984	4330	667	30306	6164	2212	408	126	N/A
Armenia	EuroB	226376	6661	475	167	3613	2117	1773	332	65	1070	218	80	26	11	N/A
Azerbaijan	EuroB	795163	23855	1670	954	12939	7581	6369	1166	369	3758	764	1448	477	308	N/A
Kyrgyzstan	EuroB	595111	17168	1250	166	9312	5456	4555	872	64	2812	572	599	197	35	N/A
Tajikistan	EuroB	870519	25144	1828	267	13638	7991	6672	1276	104	4114	837	2097	691	136	N/A
Turkmenistan	EuroB	505844	14823	1062	325	8040	4711	3943	741	126	2391	486	824	271	104	N/A
Uzbekistan	EuroB	2737750	82133	5749	3285	44549	26102	21929	4013	1272	12938	2632	4970	1638	1057	N/A
Belarus	EuroC	514996	30900	2163	1236	16760	9820	8250	1510	479	4868	990	51	17	11	N/A
Kazakhstan	EuroC	1640953	94676	6892	914	51352	30088	25117	4811	354	15510	3155	1408	464	83	N/A
Latvia	EuroC	115275	6673	484	82	3619	2121	1771	338	32	1090	222	19	6	1	N/A
Lithuania	EuroC	166177	9592	698	96	5203	3048	2545	487	37	1571	319	19	6	1	N/A
R. of Moldova	EuroC	214693	12557	902	256	6811	3991	3339	629	99	2029	413	161	53	19	N/A
Russian Federation	EuroC	8117113	487027	34092	19481	264163	154777	130036	23797	7542	76721	15604	1618	533	344	N/A
Ukraine	EuroC	2376293	139669	9980	3376	75756	44387	37167	6966	1307	22460	4568	629	207	87	N/A

ALRI – acute lower respiratory infection, SP – *Streptococcus pneumoniae*, Hib – *Haemophilus influenzae* type B, RSV – respiratory syncytial virus, FLU – influenza virus

*For viral etiologies, N/A indicates that estimates are not available at the national level at this point, due to very little available information and high degree of uncertainty of regional and global estimates.

The 2000 estimate of the proportion of pneumonia episodes that are severe was 8.6% (7.0–13.0%), and was based on 6 studies, all of them from LMIC [18]. The estimate for 2010 is based on 9 studies and brings the estimate for LMIC upward, to 11.5% (8.0–33.0%). In HIC for 2000, we did not have an evidence–based estimate for the proportion of pneumonia episodes in the community that develop into severe cases. In this current analysis, we found 9 more recent studies from HIC that show a much higher estimate of the proportion – 26.7% (20.0–46.7%). However, many of them come from hospital–based studies, where more severe episodes are likely to be clustered, and a lower threshold for severity is generally applied. Still, an increasing trend in the proportion of severe episodes in LMICs seems consistent with a higher proportion expected in HICs. Nevertheless, in an effort not to overestimate the severe pneumonia burden we elected to use the proportion of pneumonia episodes developing into severe disease from the LMIC in all HIC also.

An analysis of the prevalence of exposure to the 5 main risk factors in the year 2010 in comparison to 2000 shows that the prevalence of malnutrition declined in all LMICs from 26.9% to 21.9%, low birth weight from 15.9% to 8.8%, non–exclusive breastfeeding from 64.4% to 52.6% and solid fuel use from 65.5% to 52.2% [49,50]. The exposure to all of those risk factors fell by 20–30%, which provides plausibility to the finding that our estimate of the incidence of pneumonia fell by 25% between 2000 and 2010 in LMICs. We could not perform a similar comparison with the crowding risk factor, because of the change of the definition of crowding from “5 or more” to “7 or more” residents in the same household between surveys done in 2000 and 2010.

This study also exposed rather dramatic changes in the importance of different etiological agents along the spectrum of pneumonia episode severity. At the level of all incident episodes in a community, RSV is the most common pathogen, present in about 28.8% of all episodes, followed by influenza (17.0%), while SP (adjusted for vaccine use of both Hib and PCV) is estimated to account for only 6.9% of cases and Hib (adjusted for vaccine use) in 2.8% of cases. However, at the level of severe episodes, RSV's contribution decreased to 22.6% and influenza to 7.0%, while SP rose to 18.3% and Hib to 4.1%. Bacterial etiologies become even more important in the subgroup of the children who eventually die of the disease, with the dominant causes being SP (32.7%) and Hib (15.7%). Again, both of these estimates account for Hib and PCV vaccine use in 2010.

## DISCUSSION

Although there is seemingly more evidence used in this study than there was available in the previous studies of global childhood pneumonia morbidity [1,18], the increase in evidence is only slight: 35 studies in 1990–2010 to estimate global pneumonia incidence, in comparison to 28 studies for the period 1960–2000 [18]. This also means that the studies published between 1990 and 2000 were used to produce both estimates. However, the most recent studies (those published after 2000) are consistently showing a substantially lower incidence of community–based pneumonia than was the case historically, which implies that the burden of morbidity is steadily decreasing. This also suggests that the estimates presented in this paper maybe more closely related to the situation in the year 2000 rather than 2010, because we used the information from the previous two decades in the context of scarcity of information, and the true morbidity figures for 2010 are likely to be even smaller, ie, less than 0.20 e/cy. In addition, the decreasing trend is quite consistent with apparent improvements in risk factor prevalence, as recorded by DHS and MICS [49,50].

With more evidence, the proportion of pneumonia cases that are severe has been revised upward. For HIC, most of these estimates were relevant to children hospitalized for pneumonia, thus clustering the most severe cases, while most studies in LMICs are community–based and encompass a full spectrum of severity. Still, it appears that the increase in the proportion of severe episodes in the LMICs is a valid trend, given the high proportion in HICs (which may reflect increased proportion of parents seeking care and lower threshold for hospitalization). This finding may seem paradoxical (ie, that the proportion of pneumonia that is severe in nature is higher in high income settings that LMIC) and may be explained by a propensity to hospitalize children in HIC or by a faster reduction in pneumonia incidence at the community level than in the incidence of severe pneumonia episodes. We could speculate that improved social, economic and lifestyle conditions in many LMICs over the past decade have a rather major effect on pneumonia incidence in the whole population, while the cases that progress into severe episodes are still clustering in the areas with persistent poverty which are not really enjoying the benefits of economic growth, so it is more difficult to reduce them. If this is true, then the proportional contribution of severe episodes to all pneumonia episodes in the community is set to continue increasing over time, although both the cases of pneumonia in the community and severe cases are being reduced – but the former is being reduced at a faster pace than the latter.

It is reassuring that the etiological estimates for SP, Hib, RSV and influenza, which were based on entirely different data sets from those that were used for the estimates of pneumonia incidence, severe morbidity and mortality, and which were also conducted independently of each other, are all “fitting” into the envelopes of pneumonia incidence, severe morbidity and pneumonia deaths at the global, regional and (for SP and Hib) also at the national level [37–46]. At the level of pneumonia incidence, there is likely a multitude of etiological causes that contribute to pneumonia in the community. Therefore, at the global level, the four major pathogens combined explain about 55% of all episodes, according to our computations, but this grows to considerably more among pneumonia deaths [43–46]. The modeling effort does not reveal whether the unattributed fractions are likely from these four pathogens or from other pathogens. As an example, at the level of severe episodes, it appears that the four pathogens explain nearly 95% of all episodes in Europe and 83% in the Americas, but only 48% in Eastern Mediterranean region and as little as 39% in sub–Saharan Africa. A large ongoing project funded by The Gates Foundation – “The Pneumonia Etiology Research for Child Health (PERCH)” study – will try to explain etiology of childhood pneumonia better at the global level [25]. It is a 7–site study in LMICs, coordinated by Johns Hopkins University, to determine the etiology, or causes, of pneumonia, and the first results are expected in the latter part of 2014 [25].

All burden of disease estimates must cope with the issue of uncertainty in the data and the estimates. All the estimates of childhood pneumonia at either global, regional or (especially) national levels are inherently uncertain, for the many reasons mentioned in the beginning of this paper. The evidence to population models remains limited, and the case definitions used across studies are not all the same, yet the estimates are rather robust. What makes them plausible, if not certain, is that they are internally consistent: mutually independent cause–specific etiology estimates “fit” into the “envelopes” of total cases, severe cases and deaths; in addition, case–fatality rates between incident cases and deaths, and severe episodes and deaths, based on our model, resemble those observed in real data. This is all reassuring, but it also needs to be noted that the estimates of uncertainty (presented in Online Supplementary Document(Online Supplementary Document)) are still probably (substantially) under–estimated. This is because each and every parameter derived from a previous parameter (eg, proportion of all acute lower respiratory infection (ALRI) cases that are SP) has its own uncertainty, as do the estimates of vaccine effectiveness, risk factor odds ratios, rates of vaccine coverage, and all other parameters in the model. We typically expressed only uncertainty related to each specific parameter, without adding the uncertainty of all the previous parameters from which the new estimate has been derived. This makes the whole table in the Online Supplementary Document(Online Supplementary Document) seem more precise than it actually may be, given that the uncertainties are really large, and only the consistency between different estimates in the much bigger picture is what gives it some credibility with the current amount of evidence. CHERG aims to continue identifying new sources of published and unpublished good quality data and updating these estimates regularly with this new information and so increase the quality of its estimates towards the Millennium Development Goal 4 target in 2015 and well beyond, until preventable childhood diseases are adequately controlled and responded to everywhere in the world [57,58].
